# Pylephlebitis With Reversible Mesenteric Ischemia in a Patient With Complicated Acute Appendicitis: A Case Report

**DOI:** 10.7759/cureus.106201

**Published:** 2026-03-31

**Authors:** Marcia Mejía Velásquez, Ana M Trujillo Gómez

**Affiliations:** 1 Radiology, Universidad de Antioquia, Medellín, COL; 2 Radiology, Clínica las Américas Auna, Medellin, COL

**Keywords:** acute appendicitis, diagnostic imaging, hepatic abscess, mesenteric ischemia, pylephlebitis

## Abstract

Pylephlebitis is a rare complication of intra-abdominal and pelvic infections, occurring in the context of conditions such as acute appendicitis and diverticulitis, among others. It consists of septic thrombophlebitis of the portal vein and/or its tributary branches. Early diagnosis of this condition is crucial to prevent serious complications like sepsis and mesenteric ischemia. Contrast-enhanced abdominal CT scan is the test of choice for diagnosis and for evaluating potential complications, and the correct interpretation of radiological findings is essential to avoid unnecessary interventions. This case report describes an adult patient with pylephlebitis secondary to acute appendicitis and a rare association with mesenteric-colonic ischemia. The patient had a favorable outcome following treatment with antibiotics and anticoagulants, and the ischemia was reversible. The report emphasizes the radiological findings.

## Introduction

Pylephlebitis, occurring in 0.37-2.7 cases per 100,000 people annually, is a rare complication of intra-abdominal and pelvic infections [1]. It involves suppurative thrombosis of the portal vein and/or its tributary branches (splenic, superior mesenteric, and inferior mesenteric veins). Common causes include diverticulitis and acute appendicitis, but it can also arise from cholecystitis/cholangitis, liver abscesses, acute pancreatitis, enteritis/colitis, among others. Thrombosis occurs as a result of inflammation and a state of hypercoagulability in the venous structures adjacent to the infected area, with subsequent spread to the portal venous system [1,2].

The diagnosis of this condition is often incidental during investigations for intra-abdominal infections. Symptoms are nonspecific, typically fever and abdominal pain [3]. Radiological imaging is essential for diagnosis, with contrast-enhanced abdominal CT being the imaging modality of choice, revealing filling defects in the portal vein and affected tributaries. CT scanning is particularly useful because it not only helps determine the cause of portal vein thrombosis but also identifies complications such as abscesses, intestinal ischemia, chronic portal vein thrombosis, intestinal perforation, and peritonitis, among others [1-3].

Treatment focuses on controlling the infection with antibiotics and, depending on the cause, surgery. Delays in diagnosis and treatment increase morbidity, mortality, and complications; therefore, early diagnostic imaging and the administration of antibiotics will help improve patient outcomes. Because the clinical findings in pylephlebitis and its complications are nonspecific, it is crucial to recognize the radiological findings that allow for the differentiation of these conditions [1-5].

This case report details an adult male with acute appendicitis complicated by a periappendicular abscess, pylephlebitis, and hepatic abscesses. Pylephlebitis involved the portal and superior mesenteric veins, leading to secondary transverse colonic ischemia (a rare complication). The patient required treatment that included controlling the source of infection as well as anticoagulation (an uncommon approach in pylephlebitis), resulting in complete recovery, including resolution of mesenteric ischemia. The report emphasizes the radiological findings of pylephlebitis and its complications.

## Case presentation

A 35-year-old man with no significant medical history presented with 10 days of mild, diffuse abdominal pain and nausea. On admission, he had tachycardia and otherwise normal vital signs (heart rate 115 bpm, blood pressure 115/73 mmHg, respiratory rate 14 breaths/min, temperature 37.4°C), as well as abdominal tenderness. Laboratory tests showed leukocytosis with neutrophilia, elevated acute-phase reactants, and mild liver function abnormalities (Table 1).

**Table 1:** Laboratory test results at admission, during follow-up, and prior to discharge are listed in the respective columns. Reference values are shown in the last column CRP - C-reactive protein; ALT - alanine aminotransferase; AST - aspartate aminotransferase

Laboratory tests	On admission	On follow-up	Prior to discharge	Reference values
Hemoglobin	15.1	14.4	14.2	14-18 g/dL
Leukocytes	19.2	17.2	9.7	4.4-12 10^3^/µL
Neutrophils	16.6	15.9	9	1.5-7.26 10^3^/µL
CRP	120	110	16	<1 mg/dL
ALT	65	78	54	4-42 U/L
AST	74	82	47	0-35 U/L
Alkaline phosphatase	251	190	170	30-120 U/L
Total bilirrubin	9	3.7	2	0.3-1 mg/dL
Direct bilirrubin	6.5	3	1.8	<0.3 mg/dL

**Figure 1:**
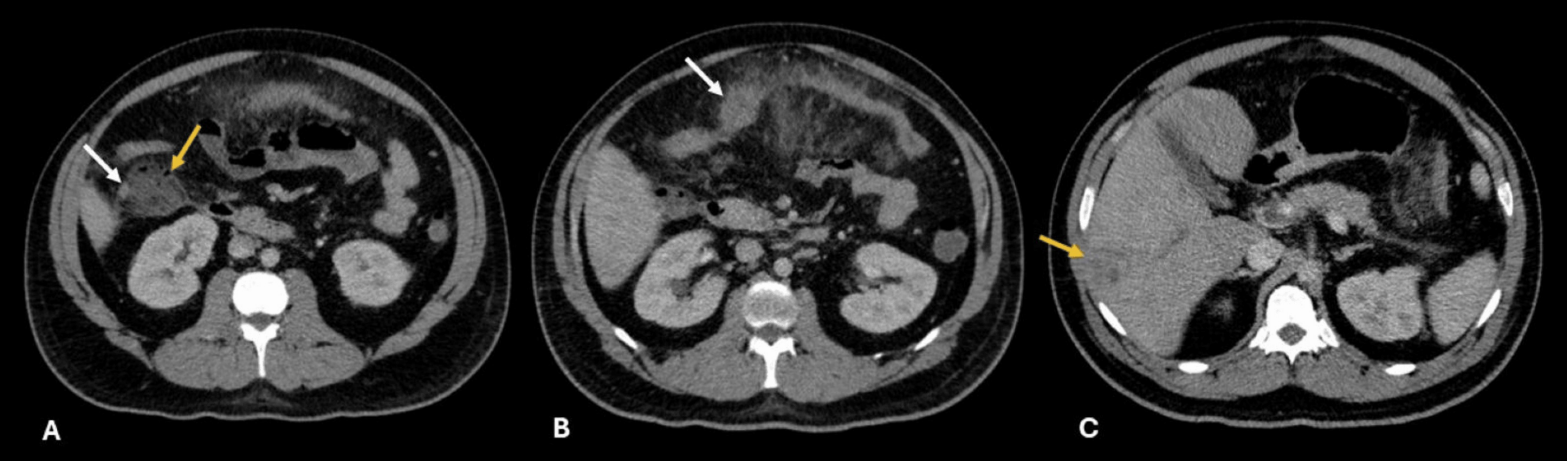
Axial, portal-phase contrast-enhanced abdominal CT A) Cecal appendicitis with wall hyperenhancement (white arrow) and adjacent abscess (yellow arrow). B) Transverse colon wall thickening with mild hypodensity (white arrow), mesenteric fat stranding, and fluid. C) Confluent microabscesses in the right hepatic lobe (yellow arrow).

**Figure 2:**
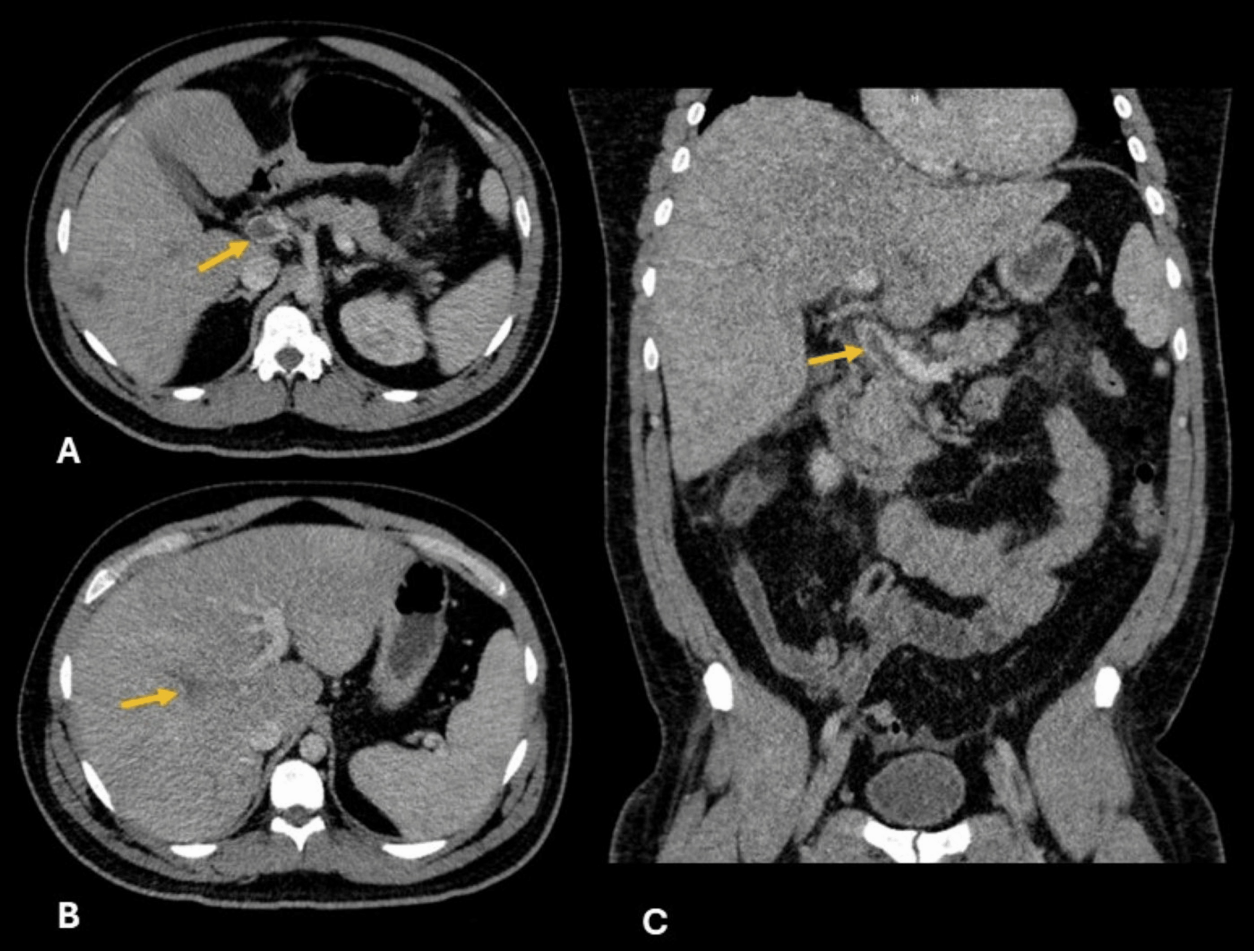
Axial and coronal, portal-phase contrast-enhanced abdominal CT Central filling defects in the main portal vein (A, yellow arrow), right portal branches (B, yellow arrow), and at the splenomesenteric junction and superior mesenteric vein (C, yellow arrow).

Contrast-enhanced abdominal CT revealed inflammatory changes in the cecal appendix with a periappendiceal abscess, portal vein thrombosis (main trunk and right branch) extending to the splenomesenteric junction and superior mesenteric vein, hepatic microabscesses, thickening and abnormal enhancement of the transverse colon (suggesting venous mesenteric ischemia), and marked mesenteric fat inflammation (Figures 1, 2).

**Figure 3:**
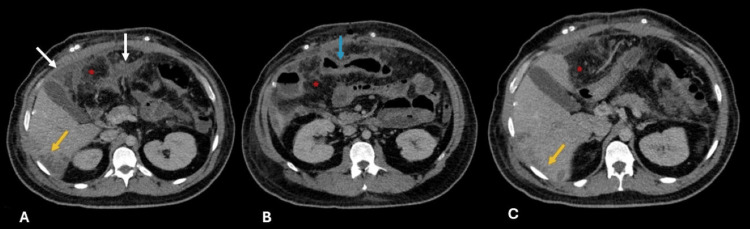
Axial, portal-phase contrast-enhanced abdominal CT A) Confluent intra-abdominal collections (white arrows) and hepatic abscesses in the right lobe (yellow arrows). B) Persistent transverse colon wall thickening with submucosal hypodensity (blue arrow). A-C) Mesenteric fat stranding (red stars) due to inflammatory changes.

Broad-spectrum antibiotics (piperacillin/tazobactam) were initiated, followed by laparotomy, which revealed: marked inflammatory changes in the greater omentum and transverse mesocolon, an area of hypoperfusion in the transverse colon (due to ischemic changes), acute perforated appendicitis with a tip abscess, and peritonitis (intraoperative images not available). Appendectomy, abscess drainage, and abdominal lavage were performed, and the abdomen was left open for later review due to severe inflammation and the possibility of residual fluid accumulation. Multidrug-sensible *Escherichia coli* was isolated from intraoperative samples and blood, but the antibiotic regimen was maintained. Anticoagulation therapy was started with low-molecular-weight heparin. Follow-up imaging showed multiple intra-abdominal fluid collections, persistent hepatic abscesses, and mesenteric/peritoneal inflammatory changes (Figure 3).

**Figure 4:**
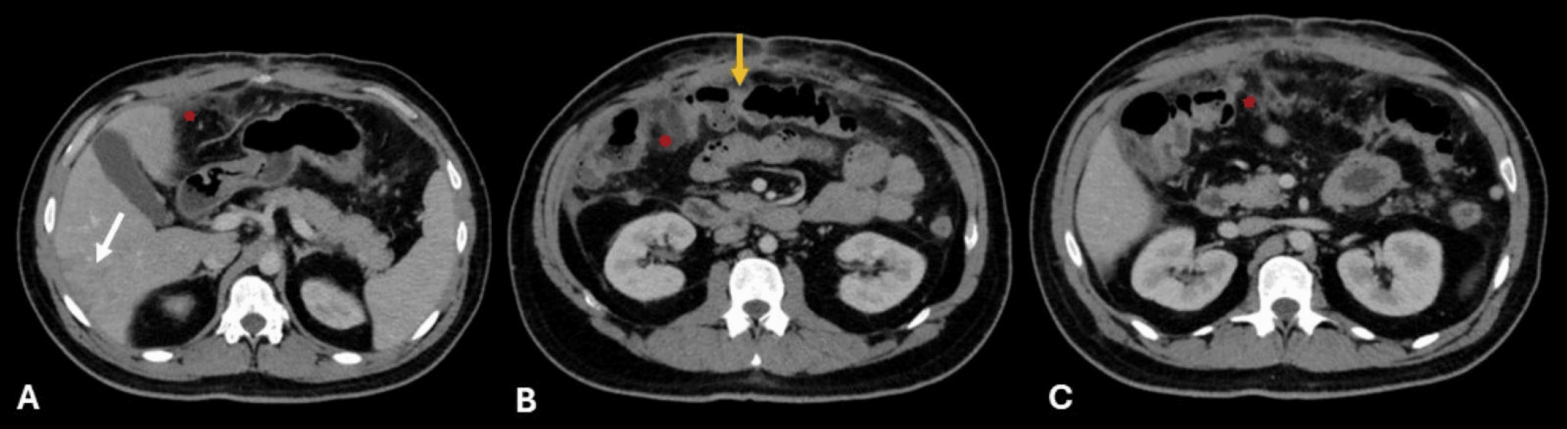
Axial contrast-enhanced abdominal CT A) Reduced size of right hepatic lobe abscesses, now with heterogeneous enhancement but without fluid (white arrow). B) Resolution of colonic wall inflammatory changes with normal wall thickness and enhancement (yellow arrow). A-C) Improved mesenteric fat stranding and laminar ascites (red stars).

Follow-up laboratory tests showed persistent leukocytosis and elevated acute-phase reactants (Table 1). A second surgical procedure involved drainage of collections and abdominal lavage. Following the second surgery, the patient's clinical condition and laboratory results improved (Table 1). Follow-up CT showed resolution of colonic ischemia, improved portal vein thrombosis, and improvement in liver abscesses and mesenteric inflammation (Figure 4).

Anticoagulation and inpatient antibiotic therapy lasted four weeks. The patient was discharged on ciprofloxacin (three weeks) and without anticoagulation, with complete symptom resolution after treatment.

## Discussion

Acute appendicitis is one of the leading causes of abdominal pain and surgical procedures worldwide (174-274 cases per 100,000 annually), affecting both pediatric and adult patients [6]. Clinical manifestations include abdominal pain (periumbilical pain migrating to the right lower quadrant), fever, anorexia, nausea, and vomiting; however, up to one-third of patients may present with atypical or insidious symptoms that are sometimes related to variable locations of the cecal appendix (retrocecal, pelvic, subhepatic, among others). Diagnosis relies on clinical findings (abdominal tenderness, leukocytosis, elevated acute-phase reactants); however, radiological imaging is increasingly used to confirm the diagnosis and rule out other causes of pain. Ultrasound is usually the imaging modality of choice in pediatric patients, and contrast-enhanced CT is used in both adult and pediatric patients when an adequate ultrasound evaluation was not possible. CT findings include increased appendix diameter (>6-10 mm), wall thickening and hyperenhancement, fluid distension, appendicoliths, periappendicular fat stranding, and ascites. Untreated appendicitis can lead to severe complications such as perforation, peritonitis, abscesses, sepsis, and pylephlebitis, emphasizing the importance of early diagnosis and treatment to reduce morbidity and mortality [6,7].

Pylephlebitis is defined as suppurative thrombosis of the portal vein or its tributary veins secondary to infections in their drainage regions [1]. It is a rare complication (0.37-2.7 cases per 100,000 people per year) of intra-abdominal and pelvic infectious diseases. The portal vein drains most of the gastrointestinal tract (except for the lower part of the rectum) and originates from the union of the superior mesenteric vein and the splenic vein. The superior mesenteric vein (SMV) drains the small intestine, ascending colon, and transverse colon, while the inferior mesenteric vein (IMV) collects blood from the left colon and upper rectum [2,3]. Pylephlebitis begins with thrombophlebitis in small-caliber veins surrounding the infected area, with subsequent migration or extension of the thrombus to the portal vein and/or its tributary branches (SMV, IMV, splenic vein) [4].

The causes of pylephlebitis vary according to age and medical history, with the main causes being diverticulitis (26.5%) and appendicitis (22%), followed by cholangitis/cholecystitis, pancreatitis, liver abscesses, gastroenteritis/colitis, post-gastrointestinal surgery conditions, inflammatory bowel disease, among others; however, in up to 11.5% of cases, the primary infection site remains unidentified [1-3]. Infections can be mono- or polymicrobial, with positive blood cultures in 70-80% of cases. Common pathogens include *Escherichia coli*, *Bacteroides spp.*, *Streptococcus spp*., and *Proteus mirabilis*, although fungal or parasitic etiologies are rare [3-5].

Pylephlebitis symptoms are nonspecific, often mimicking the underlying infection. Fever and abdominal pain are most frequent, along with malaise, diarrhea, vomiting, and, less commonly, jaundice (typically in cases of cholangitis or liver abscesses) [1-4]. Diagnosis is often incidental during investigations of intra-abdominal infections and requires radiological confirmation. Contrast-enhanced CT is preferred, allowing diagnosis of pylephlebitis, identification of the cause, and detection of complications. Ultrasound can be useful for diagnosis and follow-up, but has limitations due to operator dependence and lower spatial resolution of the gastrointestinal tract. CT findings include a central filling defect in the portal vein and/or its tributaries with perivascular fat stranding. Ultrasound reveals hyperechoic content within the portal vein and Doppler abnormalities (luminal defects on color Doppler and waveform abnormalities on spectral Doppler) [1-6]. 

Complications of pylephlebitis include chronic portal vein thrombosis with cavernomatous transformation, distant abscesses (primarily hepatic), intestinal ischemia, and portal hypertension [1,2]. 

Acute intestinal ischemia of venous origin accounts for 5-15% of cases of intestinal ischemia, involving the superior mesenteric vein in 70-95% of cases, but it may also affect the portal vein, inferior mesenteric vein, and other smaller-caliber venous branches. Association with phlebitis is very rare, and there are few reports. Contrast-enhanced abdominal CT is the diagnostic test of choice, showing central filling defects in affected veins, circumferential intestinal wall thickening (>3 mm), layered wall enhancement ("target sign"), and perienteric stranding or ascites. Venous-origin intestinal necrosis is rare because of the persistence of arterial supply and some venous drainage via collaterals; however, intestinal pneumatosis, pneumoporta, and transmural hypodensity are ominous findings that may indicate necrosis [8-10].

Abscesses associated with pylephlebitis result from hematogenous spread of the infection and most commonly involve the liver, although they can affect other organs. Management includes antibiotic therapy and drainage (percutaneous or surgical) [1-3,7].

Treatment of pylephlebitis involves controlling the infection with antibiotics and, in some cases, surgery. Anticoagulant use remains debated but is typically considered for extensive thrombosis, thrombosis progression (on follow-up imaging), persistent fever, intestinal ischemia, or hypercoagulable states. Conflicting studies exist regarding the benefits of anticoagulation, necessitating multidisciplinary decision-making weighing risks and benefits [3-6,11]. Pylephlebitis mortality ranges from 4% to 19%, with sepsis being the leading cause of death. Early diagnosis and prompt control of the infectious focus are crucial for improved outcomes [1-3].

In this case, the patient presented with insidious symptoms of acute appendicitis, which led to a delayed medical consultation. Due to the delay in diagnosis, the appendicitis was complicated by a periappendicular abscess, pylephlebitis, and hepatic abscesses. The pylephlebitis involved the portal vein and SMV, resulting in transverse colon ischemia (a rare complication). The patient required early surgical management, targeted antibiotics, and anticoagulation. The ischemic compromise of the transverse colon was reversible, with no CT or intraoperative findings indicating necrosis that would warrant intestinal resection. The patient fully recovered.

## Conclusions

Pylephlebitis is a suppurative thrombosis of the portal vein and/or its tributaries secondary to intra-abdominal or pelvic infections. It is a rare complication of intra-abdominal or pelvic inflammatory conditions. Diagnosis relies on contrast-enhanced CT, which aids in defining the underlying pathology and complications. Management focuses on controlling the source of infection and may involve anticoagulation. Early diagnosis, treatment, and radiological imaging are essential for preventing complications and serious outcomes. We presented a case of acute appendicitis complicated by abscesses and pylephlebitis, leading to mesenteric ischemia of the colon, which resolved with treatment. Emphasis was placed on key radiological findings that enabled the early diagnosis of pylephlebitis and its complications, highlighting the crucial role of CT scanning in the appropriate management of the condition.
